# HIV Care Continuum among Postpartum Women Living with HIV in Atlanta

**DOI:** 10.1155/2019/8161495

**Published:** 2019-02-14

**Authors:** Christina M. Meade, Martina Badell, Stephanie Hackett, C. Christina Mehta, Lisa B. Haddad, Andres Camacho-Gonzalez, Joy Ford, Marcia M. Holstad, Wendy S. Armstrong, Anandi N. Sheth

**Affiliations:** ^1^Brigham and Women's Hospital, Department of Internal Medicine, Boston MA, USA; ^2^Emory University School of Medicine, Atlanta GA, USA; ^3^Emory University School of Medicine, Department of Gynecology and Obstetrics, Division of Maternal-Fetal Medicine, Atlanta GA, USA; ^4^Grady Memorial Hospital, Department of Medicine, Division of Infectious Diseases, Atlanta GA, USA; ^5^Department of Biostatistics, Emory University Rollins School of Public Health, Atlanta, Georgia, USA; ^6^Emory University School of Medicine, Department of Gynecology and Obstetrics, Division of Family Planning, Atlanta GA, USA; ^7^Emory University School of Medicine, Department of Pediatrics, Division of Infectious Diseases, Atlanta GA, USA; ^8^Nell Hodgson Woodruff School of Nursing, Emory University, Atlanta GA, USA; ^9^Emory University School of Medicine, Department of Medicine, Division of Infectious Diseases, Atlanta GA, USA

## Abstract

**Introduction:**

While increased healthcare engagement and antiretroviral therapy (ART) adherence occurs during pregnancy, women living with HIV (WLWH) are often lost to follow-up after delivery. We sought to evaluate postpartum retention in care and viral suppression and to identify associated factors among WLWH in a large public hospital in Atlanta, Georgia.

**Methods:**

Data from the time of entry into prenatal care until 24 months postpartum were collected by chart review from WLWH who delivered with ≥20 weeks gestational age from 2011 to 2016. Primary outcomes were retention in HIV care (two HIV care visits or viral load measurements >90 days apart) and viral suppression (<200 copies/mL) at 12 and 24 months postpartum. Obstetric and contraception data were also collected.

**Results:**

Among 207 women, 80% attended an HIV primary care visit in a mean 124 days after delivery. At 12 and 24 months, respectively, 47% and 34% of women were retained in care and 41% and 30% of women were virally suppressed. Attending an HIV care visit within 90 days postpartum was associated with retention in care at 12 months (aOR 3.66, 95%CI 1.72-7.77) and 24 months (aOR 4.71, 95%CI 2.00-11.10) postpartum. Receiving ART at pregnancy diagnosis (aOR 2.29, 95%CI 1.11-4.74), viral suppression at delivery (aOR 3.44, 95%CI 1.39-8.50), and attending an HIV care visit within 90 days postpartum (aOR 2.40, 95%CI 1.12-5.16) were associated with 12-month viral suppression, and older age (aOR 1.09, 95% CI 1.01-1.18) was associated with 24-month viral suppression.

**Conclusions:**

Long-term retention in HIV care and viral suppression are low in this population of postpartum WLWH. Prompt transition to HIV care in the postpartum period was the strongest predictor of optimal HIV outcomes. Efforts supporting women during the postpartum transition from obstetric to HIV primary care may improve long-term HIV outcomes in women.

## 1. Introduction

Despite the availability of effective antiretroviral therapy (ART), many patients living with HIV in the United States do not achieve viral suppression, contributing to AIDS and non-AIDS morbidity and mortality and ongoing HIV transmission [1–4]. The HIV care continuum provides a framework for evaluating the quality of HIV care and identifies where drop-offs occur [5–8]. Recent recommendations for pregnancy and postpartum care mirror those of the HIV care continuum and support ongoing comprehensive postpartum care, i.e., the 4th trimester, emphasizing prenatal interventions to increase postpartum engagement in care, and the importance of a successful transition to primary care, particularly for women with chronic conditions [9]. For women living with HIV (WLWH), pregnancy provides an opportunity to optimize both the postpartum and HIV care continua. However, loss to follow-up, ART discontinuation, and the consequent lack of viral suppression frequently occur. In the US, less than 40% of WLWH are retained in care after delivery [10–19].

The majority of the over 280,000 WLWH in the United States live in Southern states, contributing to the nearly 8,500 WLWH who become pregnant annually [20–22]. Due to HIV testing and prompt initiation of ART, perinatal transmission has markedly decreased, but transmissions continue, particularly in the Southern US. The majority of infants with perinatal HIV are born to women who were diagnosed with HIV before pregnancy [23], underscoring the importance of optimizing HIV reproductive healthcare before and between pregnancies to eliminate perinatal transmissions. Further, in the Southern US, rates of postpartum obstetric visit attendance are as low as 85%, compared to the national average of 90% [24]. Few studies have evaluated postpartum reproductive and HIV outcomes for women in the South, particularly in contemporary periods when ART continuation after delivery is recommended regardless of CD4 cell count and modern regimens are used. We therefore sought to evaluate HIV and reproductive health outcomes during pregnancy and up to two years postpartum and to identify associated factors among WLWH in Atlanta, Georgia in the modern ART era.

## 2. Methods

### 2.1. Study Population and Data Collection

Data were collected by electronic medical record review of all WLWH who delivered at a large publically-funded healthcare system in Atlanta, Georgia, with at least 20 weeks gestational age, from January 1, 2011, to November 30, 2016. Supplementary data were available through August 1, 2016, from the Georgia Department of Public Health (DPH) enhanced HIV/AIDS Reporting System (eHARS) for the study population for women who did not have documented HIV follow-up within the healthcare system during all postpartum time points. Approval was obtained from the Emory University and state and institutional review boards. The study obtained a waiver of consent by the Emory University institutional review board, as it was minimal risk and did not adversely affect the rights and welfare of the subjects.

### 2.2. Sociodemographic and Clinical Variables

Sociodemographic and clinical data were collected from the electronic medical record beginning with each patient's entry into prenatal care until 24 months after delivery, including age, race/ethnicity, year of HIV diagnosis, mode of HIV infection, ART history, HIV resistance, CD4 and HIV-1 RNA (viral load) results, gravidity, parity, number of prenatal care visits, mode of delivery, gestational age at delivery, birth outcome, attendance of postpartum obstetric follow-up (within 90 days), time to first postpartum HIV care visit, and contraceptive plan and use. Subsequent pregnancy was determined for all study participants through November 30, 2016, including those that resulted in abortion or delivery at outside hospital. Transition to HIV primary care postpartum occurred at the time of the first HIV care visit or viral load measurement (excluding viral loads checked at the postpartum obstetric visit). For each postpartum HIV care visit, viral load, method of contraception, pregnancy status, ART regimen changes, adherence, and transfers of care were recorded. Additional CD4 count and viral load data were obtained from eHARS.

### 2.3. Primary Outcomes

Retention in HIV care was defined as two HIV care visits or viral load measurements, greater than 90 days apart [25]. This was determined for the initial 12 months (1-365 days postpartum) and the subsequent 12 months (366-730 days postpartum), which were defined as 12 and 24 month retention, respectively. Viral suppression was considered achieved if the last HIV RNA viral load was ≤200 copies/mL in the 12 and 24-month periods, respectively. Viral suppression or retention was required at 12 months to be considered suppressed/retained at 24 months. Viral suppression was not dependent on retention. Women with no viral load data were assigned to have a viral load value of >200 copies/mL. Upon inclusion of eHARS data, the additional women retained or virally suppressed affected outcome estimates by less than 4%. Thus, since eHARS data were only available through August 1, 2016, these data were used to estimate outcomes but not for analyses of associations, for which women who reported follow-up outside the healthcare system were excluded.

### 2.4. Statistical Analysis

In the event of multiple deliveries for a woman, the first recorded delivery was used for analysis. Descriptive statistics were used as appropriate. Bivariate analyses were conducted using t-tests, Wilcoxon rank sum, and chi-square tests as appropriate to determine the associations between the sociodemographic and clinical variables and each outcome.

Separate multivariable logistic regression models were used to determine factors associated with each HIV care outcome. Covariates reported in the existing literature were initially included in the model: delivery year, age, race, perinatal HIV infection, new HIV diagnosis, number of prenatal care visits, number of prior live births, ART use before pregnancy, CD4 count at presentation, viral suppression and contraceptive plan at delivery, postpartum obstetric visit attendance, and HIV primary care visit within 3 months postpartum. Covariates which were not predictive of any outcomes in bivariate or initial multivariable analyses (p>0.05) were removed from the final model only if removal did not significantly alter associations with other variables. Model fit was assessed by Hosmer-Lemeshow test, evaluation of residuals, and predictive ability. Analyses were conducted in SAS version 9.4 (Cary, NC). For all analyses, two-sided p <0.05 was considered significant.

## 3. Results 

### 3.1. Demographic and Clinical Characteristics

Overall, 245 pregnancies occurred in 207 HIV positive women; 44 women had 2 or more (range 2-4) pregnancies. Overall, 78% of women were African-American, the average age was 28.1 years; women had a median of one prior live birth (Table 1).

The majority acquired HIV sexually (59%); 11% were perinatally infected. Pregnancy occurred a median of 3 years since HIV diagnosis, and almost a quarter of women were diagnosed with HIV during the index pregnancy. Mean CD4 count at presentation for obstetric care was 413 cells/mm^3^, and 37% of women were receiving ART at time of pregnancy diagnosis, including 48% of women diagnosed with HIV before pregnancy.

### 3.2. Obstetric and Reproductive Health Outcomes

**Figure 1:**
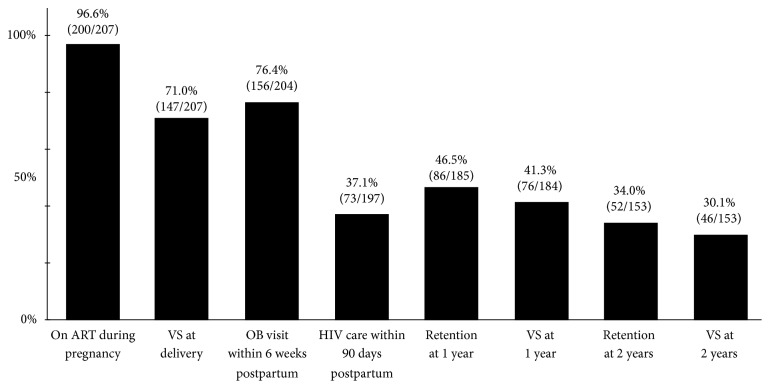
HIV and obstetric care continuum during pregnancy and two years postpartum for 207 HIV infected women.* Abbreviations.* VS: viral suppression; OB: obstetric.

Women attended a median 9 prenatal care visits, and 71% of women were virally suppressed (<200 copies/mL) at delivery (Figure 1).

About half of the women had a vaginal delivery, and 99% of the infants tested HIV negative. Overall, 62% received a contraception method by discharge, most frequently depo-medroxy progesterone (DMPA) (30%); 10% chose condoms as their only form of contraception.

Overall, 76% attended a postpartum obstetric visit. Repeat pregnancy occurred in 44 (24%) of women over a median 4.3 (Q1 2.9, Q3 5.3) follow-up years. The median time between delivery and the estimated date of subsequent conception was 358 (Q1 182, Q3 632) days; 23 (52%) occurred within 1 year of delivery, and 34 (77%) within 2 years of delivery.

### 3.3. HIV Care Outcomes

Overall, 64% of women had an HIV primary care visit within 180 days of delivery, 37% within 90 days. For women who attended a visit, the median time to HIV care visit was 124 (Q1 70, Q3 357) days postpartum. At 12 months postpartum, 86 (47%) women were retained in care and 76 (41%) were virally suppressed. Among women who were not retained in care at 12 months, 26 (26%) re-entered care and completed two visits 90 days apart between 12 and 24 months. Among women who were not virally suppressed at 12 months, 12 (11%) became virally suppressed between 12 and 24 months. Using the pre-specified criteria that retention/viral suppression was required at 12 months to be considered retained/suppressed at 24 months, 52 (34%) women were retained in care and 46 (30%) were virally suppressed at 24 months postpartum (Figure 1). With the inclusion of the statewide HIV surveillance (eHARS) data, outcomes were 102 (50%) women retained and 85 (42%) virally suppressed at 12 months postpartum, and 63 (37%) women retained and 52 (31%) virally suppressed at 24 months postpartum.

In multivariable analysis (Table 2), retention at 12 and 24 months postpartum was associated with fewer previous live births (aOR 0.73 per live birth, 95%CI 0.56-0.95; and aOR 0.71, 95%CI 0.51-0.99, for 12-month and 24-month retention, respectively), and attending an HIV care visit within 90 days postpartum (aOR 3.66, 95%CI 1.72-7.77; and aOR 4.71, 95%CI 2.00-11.10).

Retention at 12 months was also associated with older age (aOR 1.08 per year, 95%CI 1.01-1.16). Viral suppression at 12 months postpartum was associated with ART use before pregnancy (OR 2.29, 95%CI 1.11-4.74), viral suppression at time of delivery (aOR 3.44, 95%CI 1.39-8.50), and attending an HIV care visit within 90 days postpartum (aOR 2.40 95%CI 1.12-5.16). Only age was associated with achieving viral suppression at 24 months postpartum (aOR 1.09 per year, 95% CI 1.01-1.18).

Given the association between long-term outcomes and attending an HIV care visit within 90 days postpartum, we additionally examined characteristics associated with transition to HIV primary care within 90 days. CD4 count at prenatal care entry (aOR 0.998 per unit increase in CD4 count, 95%CI 0.996-1.000), lack of contraceptive provision at delivery (aOR 3.80, 95%CI 1.76-8.18), and calendar year of delivery (aOR 1.69 per year, 95%CI 1.25-2.27) were associated with transition to HIV primary care within 90 days.

## 4. Discussion

Retention in HIV care and achievement of viral suppression are necessary to maximize the benefits of ART. Despite recent improvements [26], achievement of long-term retention in care and viral suppression continues to be challenging across many US populations [6, 10]. Our study, conducted in recent years in a large-volume center in the Southern US, demonstrates that postpartum WLWH still have remarkably low 12 and 24 month retention (47% and 34%) and viral suppression (41% and 30%). Notably, the suboptimal retention and viral suppression rates observed in our study were similar to those seen in postpartum women from other US populations studied before 2012, including in Philadelphia, PA [10], Jackson, MS [27], Houston, TX [17], and Chapel Hill, NC [13]. International studies report similar challenges in South Africa [28], Ghana [29], and Uganda [30] for example, with some successful interventions reported in the literature [31–33]. Further, despite high levels of healthcare engagement during pregnancy, with an average number of prenatal care visits consistent with guidelines [34], retention and viral suppression among postpartum women in our study were even lower than those previously observed among men and women initiating HIV care in the same healthcare system, where 81% and 54% of patients were retained, and 63% and 44% were virally suppressed at 12 and 24 months, respectively. [6].

Attending an HIV care visit within 90 days postpartum was the strongest and most consistent predictor of HIV outcomes, similar to a study which used city-wide data from Philadelphia [10]. Despite over 75% women attending the postpartum obstetric visit within six weeks, less than 40% of women attended an HIV care visit within 90 days. The postpartum period is often associated with a transition of care from obstetric to HIV clinics. Fragmentation of care between the antepartum and postpartum period has previously been reported to contribute to poor follow-up among women with pregnancy-related health conditions, such as gestational diabetes and pregnancy-induced hypertension [35–39]. This loss to follow-up puts women at risk for disease progression, and puts future pregnancies at risk for increased maternal and fetal complications [37, 39].

The American College of Obstetricians and Gynecologists (ACOG) recently highlighted the importance of the postpartum period and transition to primary care in a set of new guidelines, stressing the need for an individualized spectrum of follow-up over the initial twelve weeks postpartum [9]. With 50% of pregnancy related mortality and morbidity occurring in the postpartum period, it is critical to engage women in their postpartum healthcare plan during the prenatal period. This includes identifying a primary care and other providers, scheduling follow-up prior to delivery, choosing contraception, and counseling on the effects and complications of pregnancy on postpartum physical and mental health, as well as chronic conditions, such as hypertension, diabetes, mood disorders, among others. Interventions occurring during the prenatal period have been shown to improve postpartum care for women with several health conditions, including gestational diabetes [35], pregnancy-induced hypertension [39], depression [40], intravenous drug use [41], and tobacco use [42], further supporting the notion that pregnancy and the early postpartum period (the “4th trimester”) provide an opportunity to develop and implement interventions for chronic diseases requiring close follow-up, including HIV.

By analyzing data from a single healthcare system in which HIV primary care, specialized HIV/obstetric care, and pediatric care for HIV exposed infants occurs in close geographic proximity, we showed, despite minimization of operational barriers that may be expected from co-located services [43], women in our study still experienced poor transition from obstetric to HIV primary care, similar to observations from US sites with varying structures of care [10, 12]. A recent single center report from South Carolina showed that HIV-centered prenatal care, in which HIV-trained obstetric providers provide care in a model that integrates services in one location, was associated with improved maternal virologic control during pregnancy and postpartum [44], but not improved HIV follow-up care in the 12 months postpartum, suggesting, consistent with our findings, that additional steps are likely needed across diverse care settings to improve long-term outcomes [45]. Such steps may need to also include policy changes, such as addressing insurance coverage gaps parental leave policies that particularly impact women with chronic health conditions during the postpartum period.

Temporal changes occurred within and outside our healthcare system, such as community-based interventions, streamlined clinic enrollment procedures, and enhanced care coordination between HIV and obstetric services. As such, we observed that prompt postpartum transition to HIV primary care improved over time. Nonetheless, strong associations between prompt transition and improved long-term outcomes remained even after controlling for year of delivery in our models. As has been proposed for addressing challenges surrounding transition of adolescents living with HIV from pediatric to adult care [46] and in ACOG's “fourth trimester” recommendations, a cross-disciplinary approach to integrate HIV obstetric care during pregnancy and coordinate the transition of care postpartum may thereby improve long-term HIV outcomes.

Limited evidence-based interventions exist to improve retention in care. Our findings highlight the urgent need for targeted interventions to improve outcomes in this highly vulnerable population. Previous studies have identified complex, multi-level factors associated with postpartum disengagement, such as lack of HIV disclosure, HIV-related stigma, transportation barriers, substance abuse, limited social support, and competing responsibilities such as children and work [12, 15, 27, 46, 47]. In our study, younger women and women with previous births had worse long-term retention, consistent with previous literature [17, 18, 27, 47], highlighting that a complex array of factors likely continue to contribute to poor long-term HIV care outcomes. Novel, individualized strategies are needed to identify women at the highest risk for poor outcomes and address their barriers to care.

Finally, repeat pregnancy occurred in one quarter of women, most within 1 year of delivery. While pregnancy intention was not captured in our dataset, the frequency and timing of repeat pregnancy and low uptake of highly effective postpartum contraception further emphasize the importance of integration of HIV and reproductive healthcare to improve long-term HIV care outcomes. Interestingly, contraceptive provision at time of delivery was associated with prolonged transition from obstetric to HIV primary care on multivariable analysis. This finding was likely due to increased DMPA provision at delivery for women in whom there was a high concern for loss to follow-up.

There are limitations to this study. First, as a retrospective chart review, we relied on the accuracy and scope of electronic medical records. Thus, some socioeconomic factors (ex. health insurance, housing, employment, HIV disclosure status, and partner involvement) were not consistently available. Second, this single center analysis may not be generalizable to postpartum WLWH in other settings. However, our use of statewide surveillance data, which provided rates of viral suppression and retention for the majority of postpartum WLWH regardless of location of follow-up, did not significantly alter our HIV outcome estimates and mitigates this limitation. Third, the small number of women achieving 24 month viral suppression reduced power to identify associated predictors.

## 5. Conclusion

In conclusion, we characterize a contemporary description of the HIV care continuum in a population of postpartum WLWH in a large-volume healthcare system in the South, the center of the HIV epidemic in the US, and the area with the highest number of perinatal transmissions. Even in the modern ART era, retention in care and viral suppression were low 1 and 2 years after delivery despite high levels of healthcare engagement during pregnancy and even lower than a historical control population of new and out-of-care patients in the same clinic. Prompt transition to HIV primary care in the postpartum period was the strongest predictor of postpartum HIV outcomes, suggesting that targeted interventions to engage women during pregnancy and the early postpartum period may improve long-term HIV outcomes in women. Further research is urgently needed to develop targeted interventions to improve HIV care outcomes for this population.

## Figures and Tables

**Table 1:** Demographic and clinical characteristics of WLWH in Atlanta, GA (n=207)^1^.

*Variable*	N (%) except as noted
Age, (years), mean ± SD	28.1 ± 6.2

Race/ethnicity	
White (non-Hispanic)	11 (5.3%)
African-American (non-Hispanic)	162 (78.3%)
Hispanic	14 (6.8%)
Other	20 (9.6%)

Year of delivery	
2011-2012	83 (40.1%)
2013-2014	71 (34.3%)
2015-2106	53 (25.6%)

HIV Transmission Risk Factor	
Sexual	122 (58.9%)
Perinatal	22 (10.6%)
Other (IVDU, iatrogenic)	5 (2.4%)
Unknown	58 (28.0%)

HIV diagnosis during pregnancy	47 (22.7%)

Time since HIV diagnosis (years), median (Q1, Q3)	3 (1, 8)

In HIV care prior to pregnancy	114 (55.3%)

On ART prior to pregnancy	74 (36.8%)

Number of previous live births, median (Q1, Q3)	1 (0, 2)

Number of prenatal care visits, median (Q1, Q3)	9 (6, 11)

CD4 cell count at presentation (cells/mm3), mean ± SD	412.9 ± 264.4

Viral Loads	
Viral load <1000 c/mL during entire pregnancy	85 (42.1%)
Viral load <200 c/mL during entire pregnancy	62 (30.7%)
Viral load <1000 c/mL in third trimester	150 (75.8%)
Viral load <200 c/mL in third trimester	127 (64.1%)
Viral load <200 c/mL at delivery	147 (71.0%)

ART interruption during pregnancy	133 (64.9%)

Cesarean delivery	99 (48.1%)

Perinatal HIV infection	3 (1.5%)

Contraceptive plan at delivery (mutually exclusive)^1^	
Tubal Ligation	20 (9.8%)
Hormonal intrauterine device	17 (8.3%)
Copper intrauterine device	4 (2.0%)
Implant	30 (14.6%)
DMPA	62 (30.2%)
Oral contraceptives	19 (9.3%)
Hormonal patch or ring	3 (1.5%)
Condoms alone	20 (9.8%)
None	15 (7.3%)

Contraceptive provided at time of delivery	126 (62.1%)

Attended 6 week obstetric postpartum appointment	156 (76.4%)

Subsequent pregnancy	44 (23.5%)

Postpartum transition to HIV Care	
Time (days), median (Q1, Q3)	124 (70, 357)
<90 days	73 (37.1%)
90-180 days	54 (27.4%)
> 180 days	70 (35.5%)
No postpartum HIV visit	10 (4.8%)

ART interruption after delivery	97 (64.2%)

Change in ART regimen postpartum	90 (59.2%)

Viral suppression^2^	
12 months postpartum	76 (41.3%)
24 months postpartum	46 (30.1%)

Retention in HIV care^2^	
12 months postpartum	86 (46.5%)
24 months postpartum	52 (34.0%)

*Abbreviations.* IVDU: intravenous drug use; DMPA: Depo Medroxyprogesterone acetate; IUD: intrauterine device.

^1^ Some women with a contraceptive plan did not receive planned contraception at the time of delivery.

^2^ Changes in denominator occurred because only 185 women were least 12 months postpartum, and only 153 of the women were at least 24 months postpartum, at the time of analysis.

**Table 2:** Multivariable logistic regression models^*∗*^ for factors associated with postpartum HIV care retention and viral suppression among WLWH delivering in Atlanta, Georgia, 2011-2016 (n=207).

Predictor variable	Adjusted Odds Ratio (95% Confidence Interval)			
	Retention at 12 months	Retention at 24 months	Viral suppression at 12 months	Viral suppression at 24 months
Year of delivery	1.13 (087-1.46)	1.42 (0.96-2.10)	0.92 (0.71-1.20)	0.87 (0.58-1.28)

Age in years	**1.08 (1.01-1.16)**	1.06 (0.98-1.15)	1.05 (0.98-1.13)	**1.09 (1.01-1.18)**

Number of previous live births	**0.73 (0.56-0.94)**	**0.71 (0.51-0.99)**	0.88 (0.68-1.15)	0.82 (0.60-1.12)

Perinatal HIV infection	0.31 (0.08-1.19)	0.36 (0.06-2.05)	0.32 (0.08-1.15)	ND

Number of prenatal care visits	1.06 (0.96-1.17)	1.11 (0.98-1.25)	1.05 (0.94-1.16)	1.12 (0.99-1.26)

On ART at pregnancy diagnosis	1.21 (0.58-2.52)	1.12 (0.48-2.63)	**2.29 (1.11-4.74)**	1.58 (0.69-3.65)

Viral suppression at delivery	0.81 (0.35-1.85)	1.17 (0.42-3.25)	**3.44 (1.39-8.50)**	2.24 (0.71-7.11)

Attended postpartum obstetric visit	1.82 (0.78-4.26)	1.54 (0.51-4.62)	1.54 (0.62-3.82)	1.12 (0.38-3.35)

Attended HIV care visit within 90 days of delivery	**3.66 (1.72-7.77)**	**4.71 (2.00-11.10)**	**2.40 (1.12-5.16)**	2.26 (0.96-5.35)

*Abbreviations.* ND: not determined due to small number of observations.

*∗* Separate models performed for each outcome. Covariates reported in the existing postpartum HIV care literature were initially included in each model. Covariates which were not predictive of any of the key outcomes in bivariate or initial multivariable analyses (p>0.05) were sequentially removed and excluded from the final model if removal did not alter associations with other variables.

## Data Availability

The datasets generated during or analyzed during this study are not publicly available in order to protect patient privacy. Data are available from Dr. Anandi Sheth, email: ansheth@emory.edu, 341 Ponce de Leon Ave., Atlanta, GA 30308, (404) 616-6240 (office); (404) 616-9898 (fax), for researchers who meet criteria for access to confidential data.

## References

[1] Cohen M. S., Chen Y. Q., McCauley M. (2011). Prevention of HIV-1 infection with early antiretroviral therapy. *The New England Journal of Medicine*.

[2] Mugavero M. J., Napravnik S., Cole S. R. (2011). Viremia copy-years predicts mortality among treatment-naive HIV-infected patients initiating antiretroviral therapy. *Clinical Infectious Diseases: An Official Publication of the Infectious Diseases Society of America*.

[3] Palella F. J., Delaney K. M., Moorman A. C. (1998). Declining morbidity and mortality among patients with advanced human immunodeficiency virus infection. *The New England Journal of Medicine*.

[4] Skarbinski J., Rosenberg E., Paz-Bailey G. (2015). Human immunodeficiency virus transmission at each step of the care continuum in the United States. *JAMA Internal Medicine*.

[5] (2016). *HIV/AIDS Care Continuum*.

[6] Colasanti J., Kelly J., Pennisi E. (2016). Continuous retention and viral suppression provide further insights into the HIV care continuum compared to the cross-sectional HIV care cascade. *Clinical Infectious Diseases*.

[7] Gardner E. M., McLees M. P., Steiner J. F., Del Rio C., Burman W. J. (2011). The spectrum of engagement in HIV care and its relevance to test-and-treat strategies for prevention of HIV infection. *Clinical Infectious Diseases*.

[8] Mugavero M. J. (2016). Elements of the HIV care continuum: Improving engagement and retention in care. *Topics in Antiviral Medicine*.

[9] Optimizing Postpartum Care (2018). ACOG Committee Opinion No. 736. American College of Obstetricians and Gynecologists. *Obstetrics & Gynecology*.

[10] Adams J. W., Brady K. A., Michael Y. L., Yehia B. R., Momplaisir F. M. (2015). Postpartum engagement in HIV care: an important predictor of long-term retention in care and viral suppression. *Clinical Infectious Diseases*.

[11] Bardeguez A. D., Lindsey J. C., Shannon M. (2008). Adherence to antiretrovirals among US women during and after pregnancy. *Journal of Acquired Immune Deficiency Syndromes*.

[12] Buchberg M. K., Fletcher F. E., Vidrine D. J. (2015). A mixed-methods approach to understanding barriers to postpartum retention in care among low-income, HIV-infected women. *AIDS Patient Care and STDs*.

[13] Chen J. S., Pence B. W., Rahangdale L. (2018). Postpartum HIV care continuum outcomes in the Southeastern US. *AIDS*.

[14] Mellins C., Chu C., Malee K. (2008). Adherence to antiretroviral treatment among pregnant and postpartum HIV-infected women. *AIDS Care*.

[15] Önen N. F., Nurutdinova D., Sungkanuparph S., Gase D., Mondy K., Overton E. T. (2008). Effect of postpartum HIV treatment discontinuation on long-term maternal outcome. *Journal of the International Association of Physicians in AIDS Care*.

[16] Sha B., Tierney C., Cohn S. (2011). Postpartum viral load rebound in HIV-1-infected women treated with highly active antiretroviral therapy: AIDS clinical trials group protocol A5150. *HIV Clinical Trials*.

[17] Siddiqui R., Bell T., Sangi-Haghpeykar H., Minard C., Levison J. (2014). Predictive factors for loss to postpartum follow-up among low income HIV-infected women in Texas. *AIDS Patient Care and STDs*.

[18] Swain C., Smith L. C., Nash D. (2016). Postpartum human immunodeficiency virus care among women diagnosed during pregnancy. *Obstetrics & Gynecology*.

[19] Turner B. J., Newschaffer C. J., Zhang D., Cosler L., Hauck W. W. (2000). Antiretroviral use and pharmacy-based measurement of adherence in postpartum HIV-infected women. *Medical Care*.

[20] Centers for Disease Control and Prevention (2016). *HIV Surveillance Report, 2015*.

[21] Centers for Disease Control and Prevention (2017). *HIV Among Pregnant Women, Infants, and Children*.

[22] Haddad L. B., Wall K. M., Mehta C. C. (2017). Trends of and factors associated with live-birth and abortion rates among HIV-positive and HIV-negative women. *American Journal of Obstetrics & Gynecology*.

[23] Taylor A. W., Nesheim S. R., Zhang X. (2017). Estimated perinatal HIV infection among infants born in the United States, 2002-2013. *JAMA Pediatrics*.

[24] (2018). *America's Health Rankings*.

[25] Rebeiro P. F., Horberg M. A., Gange S. J. (2014). Strong agreement of nationally recommended retention measures from the institute of medicine and department of health and human services. *PLoS ONE*.

[26] Martin D. A., Luz P. M., Lake J. E. (2014). Improved virologic outcomes over time for HIV-infected patients on antiretroviral therapy in a cohort from Rio de Janeiro, 1997-2011. *BMC Infectious Diseases*.

[27] Rana A. I., Gillani F. S., Flanigan T. P., Nash B. T., Beckwith C. G. (2010). Follow-up care among HIV-infected pregnant women in Mississippi. *Journal of Women's Health*.

[28] Phillips T. K., Clouse K., Zerbe A., Orrell C., Abrams E. J., Myer L. (2018). Linkage to care, mobility and retention of HIV-positive postpartum women in antiretroviral therapy services in South Africa. *Journal of the International AIDS Society*.

[29] Reece R., Norman B., Kwara A., Flanigan T., Rana A. (2016). Retention in care of HIV-positive postpartum females in Kumasi, Ghana. *Journal of the International Association of Providers of AIDS Care*.

[30] Obai G., Mubeezi R., Makumbi F. (2017). Rate and associated factors of non-retention of mother-baby pairs in HIV care in the elimination of mother-to-child transmission programme, Gulu-Uganda: A cohort study. *BMC Health Services Research*.

[31] Abrams E. J., Langwenya N., Gachuhi A. (2018). Impact of universal antiretroviral therapy for pregnant and postpartum women on antiretroviral therapy uptake and retention. *AIDS*.

[32] Fayorsey R. N., Wang C., Chege D. (2019). Effectiveness of a lay counselor—Led combination intervention for retention of mothers and infants in HIV care: A randomized trial in kenya. *JAIDS Journal of Acquired Immune Deficiency Syndromes*.

[33] Geldsetzer P., Yapa H. M. N., Vaikath M. (2016). A systematic review of interventions to improve postpartum retention of women in PMTCT and ART care. *Journal of the International AIDS Society*.

[34] (2016). *WHO Recommendations on Antenatal Care for A Positive Pregnancy Experience*.

[35] Bernstein J. A., McCloskey L., Gebel C. M., Iverson R. E., Lee-Parritz A. (2016). Lost opportunities to prevent early onset type 2 diabetes mellitus after a pregnancy complicated by gestational diabetes. *BMJ Open Diabetes Research & Care*.

[36] Kim C., Tabaei B. P., Burke R. (2006). Missed opportunities for type 2 diabetes mellitus screening among women with a history of gestational diabetes mellitus. *American Journal of Public Health*.

[37] Levine L. D., Nkonde-Price C., Limaye M., Srinivas S. K. (2016). Factors associated with postpartum follow-up and persistent hypertension among women with severe preeclampsia. *Journal of Perinatology*.

[38] Stasenko M., Cheng Y. W., McLean T., Jelin A. C., Rand L., Caughey A. B. (2010). Postpartum follow-up for women with gestational diabetes mellitus. *American Journal of Perinatology*.

[39] Tawfik M. Y. (2016). The impact of health education intervention for prevention and early detection of type 2 diabetes in women with gestational diabetes. *Journal of Community Health*.

[40] Matthey S., Kavanagh D. J., Howie P., Barnett B., Charles M. (2004). Prevention of postnatal distress or depression: An evaluation of an intervention at preparation for parenthood classes. *Journal of Affective Disorders*.

[41] O'Neill K., Baker A., Cooke M., Collins E., Heather N., Wodak A. (1996). Evaluation of a cognitive-behavioural intervention for pregnant injecting drug users at risk of HIV infection. *Addiction*.

[42] Lando H. A., Valanis B. G., Lichtenstein E. (2001). Promoting smoking abstinence in pregnant and postpartum patients: A comparison of 2 approaches. *American Journal of Managed Care*.

[43] Momplaisir F. M., Storm D. S., Nkwihoreze H., Jayeola O., Jemmott J. B. (2018). Improving postpartum retention in care for women living with HIV in the United States. *AIDS*.

[44] Powell A. M., DeVita J. M., Ogburu-Ogbonnaya A., Peterson A., Lazenby G. B. (2017). The effect of HIV-centered obstetric care on perinatal outcomes among a cohort of women living with HIV. *JAIDS Journal of Acquired Immune Deficiency Syndromes*.

[45] Murray Horwitz M. E., Molina R. L., Snowden J. M. (2018). Postpartum care in the united states — New policies for a new paradigm. *The New England Journal of Medicine*.

[46] Tanner A. E., Philbin M. M., DuVal A., Ellen J., Kapogiannis B., Fortenberry J. D. (2016). Transitioning adolescents with HIV to adult care: Lessons learned from twelve adolescent medicine clinics. *Journal of Pediatric Nursing*.

[47] Boehme A. K., Davies S. L., Moneyham L., Shrestha S., Schumacher J., Kempf M.-C. (2014). A qualitative study on factors impacting HIV care adherence among postpartum HIV-infected women in the rural southeastern USA. *AIDS Care Psychological and Socio-medical Aspects of AIDS/HIV*.

